# The role of the Africentric worldview in the Liberatory process

**DOI:** 10.3389/fsoc.2023.1089563

**Published:** 2023-04-12

**Authors:** Brian J. Hill

**Affiliations:** Hospitality Management, Graham School of Management, Saint Xavier University, Chicago, IL, United States

**Keywords:** African-American, Africentric, Liberatory process, graduate education, Selfethnic

## Abstract

As the Black Lives Matter movement brings increasing awareness to systemic racism in American society, the Eurocentric worldview and intellectual racism nonetheless remain prevalent. However, opportunity to engage with Africentric knowledge that could help counter the focus on Eurocentric worldview among adults remains limited in graduate courses. When such opportunities do arise, however, they are powerful and transformative. This paper conducts a case study of four students of African descent enrolled in graduate programs that engaged with Africentric knowledge. The results demonstrate that such experiences can create a powerful Liberatory transformation. The five step Liberatory process is a cognitive awakening and a discussion of the implications for practice and research are presented as well.

## 1. Introduction

In recent years, consciousness of racial identity issues has significantly increased in many regards. One principal manifestation of this heightened awareness is the emergence of the Black Lives Matter movement and the resultant open acknowledgment of systemic racism in many aspects of contemporary American society (Hargons et al., 2017). However, even as the nation reckons with systemic racism in its more tangible forms, systemic intellectual racism remains more challenging to address (Dei and Linton, 2019). In particular, systemic intellectual racism is a product of the American educational system's foundation on a Eurocentric worldview that does not ascribe appropriate agency to African persons (McLeod et al., 2019). This blind spot in the educational system extends to adult education, a particularly troubling fact given the comprehensive and reflective nature of education for students who have already matured into adulthood.

When adult education meaningfully engages with racial identity, it can create a transformative, so-called “Liberatory” experience. This idea emerged in the field of Black psychology, for example, in the work of Grills et al. (2018), who reviewed the work of the Association of Black Psychologists' work through the development of models of psychological practice, theory, and strategies grounded in African-centered and Black psychology. Such efforts to design an educational experience that engages with students' racial background and fosters racial identification can make a beneficial impact, in terms of both overcoming existing intellectual blind spots as well as helping students of African descent gain a valuable understanding of their unique sociohistorical background (Kelly and Patrice, 2019). However, when adult education of people of African descent fails to incorporate an African-centric (or Africentric) worldview, the experience fails to provide adequate value for Black students and might reinforce existing intellectual blind spots. This, in turn, deprives people of African descent of an important chance to engage with their own heritage and kinship with the broader African diaspora.

Although somewhat dated, the research reviewed in this study demonstrates that, even 15 years ago, exposure to Africentric knowledge in the context of adult education could create a transformational, Liberatory experience for people of African descent. This lesson has still not yet been learned by the adult education field or its practitioners. Therefore, in light of the ongoing awakening regarding systemic racism in the United States (US), the results of this 2005 study take on new meaning and importance, indicating a way in which adult education could be reshaped to better incorporate the Africentric perspective. This study explores how being introduced to a foundation of Africentric knowledge influenced four graduate students from Chicago, Illinois in 2005. The results also illuminate a path forward for adult education.

## 2. Materials and methods

### 2.1. Methodology and design

Given the purpose of this study—to examine the experiences of doctoral students of African descent—a qualitative methodology was the most appropriate approach to conducting this research. Qualitative research is descriptive and exploratory, serving as an ideal means for examining phenomena that have been relegated to the periphery of existing research (Merriam and Tisdell, 2015). Because a qualitative method is open-ended, it accommodates the exploration of all potentially present perspectives and perceptions within a population of interest (Merriam and Tisdell, 2015). This makes the qualitative lens ideal for exploring the educational experiences of doctoral students of African descent and investigating the Liberatory, transformative processes that they might have undergone. This study is deeply subjective in nature as well as entirely exploratory, making it innately qualitative in nature. Further, I acknowledge my background affected the study to some extent; however, my experience also prove to be a significant resource in making sense of the literature on the topic.

This study employed a qualitative research design employed based on a case study approach. When conducting a case study, the researcher examines one or more specific cases in order to draw conclusions about the phenomenon as a whole as well as collect contextual information (Yin, 2017). In this case, the study was concerned with the contextual aspects of the participants' experiences. Moreover, the specific case under study is relatively unique in the adult educational milieu.

The theoretical framework for this study was critical race theory, which focuses on the lived experiences of ethnic minority communities in relation to race as well as race relations (Delgado and Stefancic, 2001). Researchers have noted that critical race theory can be utilized in evaluating the different ways in which racism and race indirectly as well as directly influence ethnic minorities (Yosso, 2005). Within the framework of critical race theory racism is viewed as both an individual and group level phenomenon that works on multiple levels, including systematic and institutional. Critical race theory considers how the social constructed nature of race results in racial inequity, prejudice, and subordination, which in this context concerns the marginalization of Africentric knowledge thorough excessive systematic and institutional focus on Eurocentric worldview.

### 2.2. Population and sample

The population chosen for analysis was doctoral students of African descent. The study was temporally contextualized by being performed in the specific location of Chicago, Illinois in 2005, before the Obama era. Within this broader population, the target population was doctoral students who graduated an experimental adult education graduate program that integrated the Africentric paradigm into the curriculum. At the time of the study, there was no broad focus on racial identity development in the field of adult education, so it was crucial to recruit students from graduate programs that utilized an Africentric paradigm.

From the entire student population, four participants were recruited to offer their insights to the researchers conducting this study. Given the intensely subjective nature of the research, we will identify each participant individually under their chosen pseudonym. More in-depth information regarding these four participants is presented in the Results section. The two men, Mufasa and Dr. A, and two women, Sankofa and Sunshine, who comprised the sample were previously adult education students who graduated from doctoral programs that utilized an Africentric paradigm. The participants all came from the midwestern or the southern regions of the US and were members of the African diaspora.

### 2.3. Data collection

Data collection for this study rested primarily upon the use of qualitative interviews with the participants. These interviews examined how the Africentric knowledge base impacted each participant affectively, cognitively, and behaviorally. Semi-structured interviews, which were the primary method for gathering qualitative data, enabled the researcher to understand the participants' opinions and perceptions (Kallio et al., 2016). The semi-structured interview is flexible yet structured; a structured interview guide and sample questions ensured that each interview captured the essential points and provided the freedom to ask follow-up or probing questions to fully understand key issues (Kallio et al., 2016).

In addition to conducting the interviews in person, the fact that African-American researchers were interviewing African-American participants was key. It is the researchers' view that Africentric research conducted by Africentric scholars yields a sense of openness and honesty among the research participants. We believe that it was necessary to “meet and greet” the participants in person rather than hold impersonal phone interviews to facilitate comfortable exchanges. The in-person interviews were audio recorded and transcribed with the assistance of a professional transcription company to ensure accuracy and completeness.

Participants were located by reviewing the adult education graduate programs that utilized an Africentric paradigm and recruiting individuals who had graduated from these programs. Using this approach, the potential participants who met the inclusion criteria were identified and sent a letter requesting their participation. Those participants who expressed interest were called or emailed to confirm their interest and schedule a meeting. Upon confirmation of scheduling, each research participant was sent an informed consent document to formalize the agreement and confirm that participation in the study was voluntary and confidential. Most participants chose their home as the interview setting. As each participant showed great interest in our and support for our efforts, we offered to update them on the progress of our study as a means of maintaining an ongoing personal connection.

### 2.4. Data analysis

In this study, the traditional methods of qualitative analysis, such as qualitative thematic analysis, were set aside in favor of an analytical approach based on the study's theoretical underpinnings. The analytical approach provides a theoretical basis for describing the Liberatory process of transformation from a Eurocentric to an African-centered worldview. First, the analysis drew on the concepts discussed in Woodson's (1933) The Mis-education of the Negro to understand, describe, and articulate the research participant's experience of intellectual racism in their formal educations whereby they were misinformed or ignorant about their intellectual and sociocultural history. In addition, the concept of the self was understood using Du Bois's concept of “The Dilemma of the Double Consciousness” (Du Bois, 1903). The concept of Liberation was a key analytical tool for understanding the process of becoming free from the ignorance of their intellectual and sociocultural history and the transformation in their worldview as they learned about their intellectual and sociocultural history. Finally, the Nguzo Saba or Seven Principles—Umoja (unity), Kujichagulia (self-determination), Ujima (collective work and responsibility), Ujamma (cooperative economics), Nia (purpose), Kuumba (creativity), and Imani (faith)—were used to determine how the Liberatory transformative process resulting from the study of the Africentric intellectual knowledge base manifested in their lives affectively, cognitively, and behaviorally. The results of this Africentric analysis follow.

## 3. Results

Based on this study's Africentric analysis, five key stages in the Liberatory process emerged. These five stages are detailed later in this section. First, however, a description of the participants is presented.

### 3.1. Participants

As noted above, the sample in this study included two men and two women for a total of four participants. Two were from the Midwest and two were from the South. One participant was born in Africa, while the rest were born in the US. Each participant is introduced by their pseudonym, as follows:

*Mufasa* is an African-American male adult educator from a large city in the Midwest. He is very active in the African-American community, working for community-based organizations and serving on community boards for organizations that assist people of African descent seeking employment opportunities.

*Sankofa* grew up in an East Coast city and has been working in higher education for over 20 years. Prior to her enrollment into graduate study, she held a position in the area of higher education academic skills and assessment. She now serves as an upper-level administrator at a southern historically Black college, where she provides culturally relevant programming to students of African descent.

*Dr. A*. was born was educated in Africa during his formative years. He now lives in a Midwestern city and works as a community college professor teaching the Africentric intellectual knowledge base. Dr. A. also serves on community boards and participates in programs that promote unity, collaboration, and community involvement.

*Sunshine* is a female African-American adult educator with roots in the southern part of the US. She is currently teaching at an elementary school in a southern city dedicated to promoting knowledge of the Selfethnic to African-American youth.

### 3.2. Stage I: pre-sense of selfethnic

As the name implies, this first step represents the starting point of the Liberatory process. In the context of this study, this stage was conceptualized to articulate the participants' perceptions of their own racial/ethnic group membership prior to enrollment in their respective graduate programs. One participant, Mufasa, described a strong sense of racial identity at this point, one borne of prior graduate education and civil rights engagement. Mufasa contrasted his own feelings to those of other Blacks, who he felt lacked this strong sense of racial identity. This might be illustrated by the perspective of another participant, Sankofa. Before her graduate studies, she was not only less confident about her own racial identity, but also that of her group membership. Her negative feelings were associated with the term “minority”, which she viewed as a descriptor of not only the percentage of the population that Blacks represented, but also as an indication of their lower status and value.

Despite being born in Africa, Dr. A. did not describe a strong sense of racial identity prior to his graduate studies. In this sense, his experience helped to dispel the myth that if a person is born in Africa, they naturally understand their own racial identity, inclusive of Selfethnic knowledge. Sunshine also expressed a weak estimation of her identity, noting that it was “Very weak…I didn't consider myself as much of anything, because, prior to my graduate study, I knew that I was an African descent person, didn't put much value to it”.

### 3.3. Stage II: summoning to selfethnic awareness

Given that the pre-awareness stage was essentially a starting point, the step of summoning Selfethnic awareness represents the next meaningful step in the Liberatory process. This step corresponds to the participants' introduction to the Africentric intellectual knowledge base as a part of their curriculum. As highlighted in the pre-awareness step, part of the Liberatory process involves being freed from one's own negative self-image (Dhaliwal et al., 2020). Despite his head start in terms of Selfethnic identity, Mufasa was no less engaged at this stage, noting that, “It was a blessing for me to be able to again pursue this school of thought and be nurtured and be encouraged…it was sort of the natural thing for me to gravitate toward the Afrocentric paradigm.” In this sense, Mufasa's prior experiences strengthened his engagement with the Africentric knowledge base.

For those without the benefit of such prior experience, however, this step could be more emotional. Sankofa described the realization as follows, “I realized upon being introduced that there was a lack of knowledge on my part and I didn't know anything about my own history, that it had been left out of the textbooks, it had been left out of my development in high school, grammar school, high school, undergraduate and college, masters in college.” The realization that she was finally able to study these issues was emotionally powerful. For Dr. A., the ability to engage with this content was an opportunity to seek answers to long-held questions. He discussed his inability to answer questions about Africa that he should have been able to answer as a born and bred native African. Furthermore, Dr. A. viewed his decision to pursue this course of study as one of mutual benefit to his understanding of both America and Africa. Finally, Sunshine described the experience as finally connecting her racial identity to something more significant. She previously thought that she knew what it meant to be of African descent, but did not relate that feeling to any specific information, which was changed as a result of her encounter with the Africentric knowledge base.

Selfethnic awareness is a cognitive encounter in which a person of African descent acknowledges an awareness of a sociocultural history that places them within the African diaspora. As a result of exposure to the Africentric knowledge base during graduate studies, a person of African descent makes a cognitive correlation between themselves and their racial group (Stanger, 2018). This is key to the Liberatory process.

### 3.4. Stage III: immersion in the search for selfethnic knowledge

Because the participants' course of study necessarily led to an immersion in the Africentric knowledge base, their Liberatory process should necessarily pass through the third step, that of immersion in the search for Selfethnic knowledge. For Mufasa, a key aspect of this immersion was being able to engage with not only the written word, but also with the spoken word that was shared between him and other members of the diaspora traveling their own paths, gaining knowledge and enrichment. His immersion did not change his Selfethnic identity, but instead strengthened it. “It was strong going in and it was stronger coming out… It took it to a different level and…made me a little stronger, a little more committed, and a little more grounded—a lot more grounded, in fact—in terms of broadening my knowledge base.” Similarly, Sankofa was not content with merely reading as she became immersed in the search for Africentric knowledge and identity—she wanted to see, feel, touch, and interact with the concepts she encountered. This prompted her undertaking a pilgrimage to Africa, which caused a profound change in her self-concept: “I fell in love with it, I fell in love with my brothers and sisters because I am because you are and you are because I am. Just like when I greeted you today, it's like I've always known you, you're not a stranger to me.”

Dr. A.'s experience was a mirror of Sankofa's. Rather than going to Africa to pursue immersion, his immersion consisted of familiarizing himself with the US context and the existence of an African identity therein. Dr. A. referred to his graduate study in the Africentric intellectual knowledge base as a journey and a voyage of discovery in which he became grounded in the Africentric knowledge base, which centered him and formed a foundation from which he would be able to tell his story. Finally, Sunshine's immersion represented an opportunity for her to understand and contextualize the teachings passed down from her family that were previously unrelated to her identity. When Sunshine began her graduate studies, she did not consider herself much of anything; however, immersion in the Africentric knowledge base profoundly shifted that perspective. Overall, immersion in the search for Selfethnic knowledge encompasses an affective impact in which a person of African descent becomes energized and validated by connecting or reconnecting to the Selfethnic knowledge inherent in the Africentric intellectual knowledge base.

### 3.5. Stage IV: internalization of selfethnic knowledge

The next step on the Liberatory path is internalizing Selfethnic knowledge. Internalization reflects the fact that immersion is insufficient; at this stage, someone must take up the knowledge and truly make it their own. In Mufasa's case, this involved transforming the Selfethnic concept so that it would align with the entirety of the African diaspora. “I think one of the…most important things that I got was to look at Africans of the diaspora, which was key to understanding our situation, and when you say ‘looking at the racial group,' I kind of broadened my view beyond Africans in America.” For Sankofa, this involved a shift toward a more Africentric worldview. In contrast to her prior identity as a so-called “minority”, she stated, “I know I don't consider myself a minority. I consider myself a producer of knowledge and not a consumer… I know that I gave birth to the men and women that are going to go out there and affect change and I know that I can help my people, and that's more meaningful to me than how old I am and…as long as I have breath in my body, I will be trying to do that in some way.”

For Dr. A., the internalization of Africentric knowledge involved acquiring tools with which to challenge the dominant ideologies of other people He noted, “So this voyage of discovery…sometimes…gives you the things that you always take for granted, that you thought you know enough to say, well that's not good enough. […] [D]oing the program gave me, in a sense, that we need to…move with this, we need to really challenge Eurocentric[ism], you know, squash them that took away…what we were supposed to be taught.” Sunshine described her internalization most passionately, claiming that it had transformed her into a different person. She discussed how racism impacted her differently because of who she now was and stated, “It can't impact me. No, it wasn't the case prior to my study, I held a lot of bitterness. I think with the paradigm shift, when I learned more about…my roots, I learned more about the traditions, the Nguzo Saba, I did learn a lot about that.” As a result, she no longer internalized the way others perceived her. All of the participants contextualized knowledge relative to Africentric intellectual traditions. Each made the affective and cognitive delineation between the “self” and the Selfethnic, and their thoughts, actions, and beliefs were transformed. They differed most in how these changes were reflected behaviorally.

### 3.6. Stage V: selfethnic racial identity

The final stage of the Liberatory process represents its culmination—the establishment of a Selfethnic racial identity. Mufasa expressed the empowering nature of such an identity: “It's who you are. It's what makes you a member of a group. And there's a number of elements involved in it, of course. And if you feel good about yourself, I think that you would kind of naturally gravitate toward Afrocentricism.” This manifested as a greater commitment to his work in public service and other related fields. These behaviors and commitments are directly aligned with the Africentric principles within the Nguzo Saba. For Sankofa, the key was the development of a collective consciousness. She never spoke only of herself relative to any topic; instead, everything that she said exhibited the collective consciousness that she absorbed from Africentric intellectual traditions. This was expressed straightforwardly as, “I am an Africentric scholar, because I've been transformed, because the light is on.” Furthermore, Sankofa addressed the actions she took to provide the necessary educational elements required to achieve an African-centered worldview and linked her values as a professional educator with the personal values that she shares with her own children.

For Dr. A., Selfethnic racial identity manifested as follows: “I see a different long-term strategic mission that I need to do. I need to be much…broader, much more inclusive. […] I want people to ask me, well, what do you mean ‘activist educator'? You know, I want others to start thinking, you know, [an] activist…is a troublemaker.” This reflects an understanding that the problems facing members of the African diaspora are similar, regardless of geographic location, and that we need to address them collectively. In addition, Dr. A. spoke of the difficulties of being challenged on what you know about your own history and culture and how he is now challenging others to gain Selfethnic knowledge. Finally, Sunshine expressed that, “My liberation, I gained insight about who I was through that doctoral program. I don't need a doctorate to get a job in an elementary school, middle school. I'[ve] already surpassed that… [W]hat did I learn being a doctoral student, it's yours to have, there is no reason why you can't have it.” This reflects an understanding of the importance of how the graduate program changed her as a person more than as a professional. For Sunshine, the statement “it's yours to have” means that there is no limitation on one's potential. Sunshine equates love of self with her knowledge of the Selfethnic. Taken together, these insights lead to the conclusion that deep wisdom and understanding of the Selfethnic is the highest level of cognition that a person of African descent might experience during the Africentric transformative Liberatory process of self to Selfethnic.

## 4. Discussion

To understand the need for a Liberatory process derived from engagement with African knowledge, it is important to begin from the standpoint of understanding the prevalence of the Eurocentric worldview in the US and elsewhere. According to Nathan Ron, one's “worldview” is their perspective on how humans think, behave, and act in the world (Ron, 2019). A Eurocentric worldview ascribes emphasis on individualism, material possessions, wealth, domination, and oppositional dialecticism (e.g., good vs. evil or right vs. wrong; Muradian, 2019). These characteristics of the Eurocentric worldview, which evolved from a long philosophical tradition with roots in ancient Greece and Rome, are deeply ingrained throughout Western society and reflect a European historical context (Akpan and Odohoedi, 2016). The Eurocentric worldview is not, conceptually speaking, either right or wrong, but, rather, a perspective appropriate for Europeans and those of European descent (McLeod et al., 2019).

Given that the appropriateness of Eurocentrism is defined in terms of Europeans and their descendants, it is appropriate to define a distinct, parallel worldview centered on Africa and those of African descent (Nwoye, 2017). According to Appiah-Kubi and Aabaa (2019), viewing the world from an African-centered position enables one to see cultural activities, social interactions, political situations, and other phenomena from an orientation that prioritizes the position and interest of African peoples. One who ascribes to an African-centered worldview emphasizes the group, cooperation, and spirituality (Schiele, 2017). A characteristic African-centered worldview views wealth as equivalent to well-being, sees beauty as measured by behavior, and strives to co-exist with nature rather than conquer it (Marimba, 2017). The most well-known term to represent an African-centered worldview is “Afrocentricity”, as defined by Asante (2003):

A mode of thought and action in which the centrality of African interest, values and perspectives predominate. In regards to theory, it is the placing of African people in the center of any analysis of African phenomena, in alignment with the theoretical framework of the study, critical race theory, which focuses on the experiences of ethnic minorities (Delgado and Stefancic, 2001). Thus, it is possible for anyone to master the discipline of seeking the location of Africans in a given phenomenon. In terms of action and behavior, it is a devotion to the idea that what is in the best interest of African consciousness is at the heart of ethical behavior. Finally, Afrocentricity seeks to enshrine the idea that blackness itself is a trope of ethics. Thus, to be black is to be against all forms of oppression, racism, classism, homophobia, patriarchy, child abuse, pedophilia and white racial domination (p. 2).

However, this study takes a slightly different approach—the Africentricity or Africentric worldview approach. The Africentric worldview is similar to the Asante's Afrocentric perspective in terms of its emphasis on an African worldview and African cultural values, but it differs by focusing on (a) persons of African descent as agents in their own contexts and (b) the application of an African worldview to the specific context of adult education. The latter is significant because, even today, the adult education discipline remains largely bereft of African perspectives. In addition, Africentricity underscores the interconnectedness of members of the African diaspora.

Given that many people share both African and European ancestry, the Africentric worldview is not, and need not be, the only worldview held by those of African descent, and yet the Africentric worldview nonetheless often holds a special significance for such persons. Africentrism encompasses not only a view of the world in which members of the African diaspora are actors and agents in their experiences, but also the knowledge that a positive image of the “self” exists and that the “self” is an intrinsic characteristic positively connected to race (Boutte et al., 2017).

A key component of Africentrism is the Nguzo Saba, which reflects “the seven African Centered, transcontinental and transgenerational values/principles: Nia (Purpose), Imani (Faith), Umoja (unity), Kujichagulia (selfethnic determination), Ujima (collective work and responsibility), Ujamma (cooperative economics), and Kuumba (creativity)” (Colin, 1988, n.p). The Nguzo Saba, as described by Maulana Karenga, identifies the cultural behaviors indigenous to members of the African diaspora that are essential to an Africentric consciousness (Karenga, 1989). It reflects culturally grounded beliefs and principles. These concepts are a central underpinning of the present study.

Another key concept in this study is Assante's notion of the “Selfethnic,” whereby African peoples are positioned at the center of any analysis of African phenomena. This key concept denotes an analysis that leads to “Selfethnic” awareness by focusing on African people as subjects rather than objects (Colin and Scipio, 1989). Scipio Colin, who proposed the Selfethnic liberation, characterized it as a process to “establish principles that will liberate them from the destructive effects of socio-cultural and intellectual racism” (p. 16). Engaging the world through an Africentric worldview can, for those of African descent, give rise to a “Liberation” that places people of African descent at the center of the related analysis (Cook, 2019). This notion is central to understanding African-American racial identity as it accommodates an agent and actor analysis of all phenomena, counteracts the negative impact of intellectual and sociocultural racism, and therefore provides a path to the transformation of the “self” into the “Selfethnic” (Howard, 2009).

To understand this Liberatory process, it is necessary to first define Liberation. For this purpose, Liberation is taken to mean freedom—freedom of the body, spirit, and mind. More importantly, this freedom creates space for new concepts and ideas that are in the best interest of the individual as he or she relates to the collective (Asante, 2020). The Liberatory process of engaging with the Africentric knowledge base frees a person—one who would be better served by an Africentric worldview—from a Eurocentric worldview (Dalmage and Martinez, 2020). This Liberatory process as it manifests in graduate adult education programs is the focus of the present study. In this context, the Africentric knowledge base refers to the accumulated knowledge that has been established from examining the world through an Africentric worldview (Nwoye, 2017). In recent years, this knowledge base has become increasingly expansive and yet, within the context of the African diaspora, remains underutilized in many respects.

Through an Africentric analysis, this study concluded that the Liberatory process of studying Africentric knowledge at the graduate level consists of five steps. It begins with the pre-sense of the Selfethnic. At this stage, most African graduate students had not yet experienced or engaged with Africentric ideas, but even those who had would further develop their identities. The Liberatory process transcends externally imposed ideas; therefore, it encompasses breaking free of negative self-conceptualization. The intent of this study was to identify the elements of an Africentric Liberatory transformative process relative to the development of an African-centered racial identity that demonstrates a paradigmatic shift from a Eurocentric worldview to an African-centered worldview. The steps of the Liberatory process that were discovered included the summoning of a Selfethnic awareness, immersion in the search for Selfethnic knowledge, the internalization of Selfethnic knowledge, and finally, the establishment and actualization of Selfethnic knowledge. This Liberatory process has important implications; it is now more important than ever to apply these ideas to the realm of contemporary adult education.

### 4.1. Implications

The findings of the study addressed a gap in the literature presented due to American educational system's foundation on a Eurocentric worldview that does not ascribe appropriate agency to African persons (McLeod et al., 2019). This blind spot in the educational system extends to adult education, a particularly troubling fact given the comprehensive and reflective nature of education for students who have already matured into adulthood. Broadly speaking, the results of this study demonstrate how, even in an era of lower racial awareness and activism than today's, simply being exposed to Africentric knowledge can be a powerful and transformative experience. The participants in this study gained knowledge of the Selfethnic and experienced a paradigmatic shift to African-centered values and beliefs. As was previously established, deep wisdom and understanding of the Selfethnic is the highest level of cognition that a person of African descent can experience during the Africentric Liberatory transformative process of self to Selfethnic. The person of African descent sheds Eurocentrically held values and beliefs and embraces African-centered values and beliefs. The person of African descent becomes a living example of the legacy, hopes, dreams, and knowledge of their ancestors, with a chosen commitment to serve as an agent and actor in the mission of extending Selfethnic Liberatory transformation to all members of the African diaspora. The values and beliefs exhibited in the behavior of a person who has been impacted by Selfethnic knowledge demonstrate a commitment to liberating other members of their race through their daily lives and work, as reflected in the Nguzo Saba (Lateef and Anthony, 2020).

In this sense, the practical implications of this study reflect the fact that exposure to the Africentric knowledge base at the graduate level precipitates the development of Selfethnic racial identity. This Selfethnic identity, in turn, manifests as Liberation and engenders a practical transformation, even for those who started with some degree of an Africentric perspective. However, for those lacking such a perspective, the transformation was deeper and more impactful. This transformative process was portrayed as entirely positive by all of the study's participants, creating empowerment and boosting their sense of self and connection to the African diaspora. Given the currently increased level of racial awareness, acknowledgment of systemic racism, and desire for transformative change, it seems likely that applying the Africentric approach evinced in this study could form the basis of an even more profound Liberatory experience.

Therefore, based on these results and combined with contemporary society's current quest for racial justice, the Africentric knowledge base should be presented within all graduate adult education courses. Such inclusion can serve to empower not only those of African descent but also offer a more balanced perspective to all graduate students, regardless of racial identity. The results of this study reflect the need to include information relative to African-American racial identity in all future adult developmental theory handbooks. Furthermore, such knowledge should be included in pre-graduate-level education as well.

In contextualizing the results of this study, it is important to emphasize that the data were collected in 2005, when issues of Africentricity were less at the forefront than they are today. At the time the study was conducted, the adult education program that the participants attended was highly experimental. Subsequent events, such as the election of President Obama (Sikanku, 2020) and the popularity of the movie Black Panther (Babb, 2020), have caused Africentricity and Selfethnic identification to become more mainstream than when the interviews were conducted. At the same time, however, the rise and ongoing prevalence of the Black Lives Matter protests provides convincing evidence that issues surrounding racism, intellectual and otherwise, are far from resolved (Hargons et al., 2017). On the contrary, systemic racism in the US remains ubiquitous and increasingly problematic. In adult education, this continues to be manifested in the lack of Africentric content, a shortcoming that should be immediately addressed. For these reasons, the results of this study continue to be relevant. If introduction to the Africentric knowledge base was effective for giving rise to a Liberatory experience in 2005, then this approach to combating the effects of systemic racism in adult education holds great promise for its application today.

### 4.2. Limitations

There are several limitations present in this study. The first is its small sample size. Although small sample sizes are typical in qualitative research and the population under study was limited because of the scarcity of Africentrically focused graduate programs, four participants still does not constitute a representative sample. Instead, the participants of this study should be taken as a proof of concept—proof that engagement with the Africentric knowledge base can and does create a Liberatory process for students of African descent. Therefore, although the recommendations provided above are somewhat extensive, they should be understood in the context of this limitation. Secondly, the study was limited in that the participants hailed from only two geographic regions of the US, the Midwest and the South—though all were studying in Chicago. However, these limitations do not indicate a lack of participant diversity in other regards. On the contrary, the participants hailed from a wide array of backgrounds, including civil rights activists and African immigrants.

Another limitation of the present study is that the data were collected in the past. Hence, although they still have important implications, it is important to supplement these conclusions by conducting additional contemporary data collection. In addition, this study might have been limited by the biases and perspectives of the researchers, who are themselves of African descent. To guard against such biases, the results and the interpretations are supported by quotations from the study's participants wherever possible. It is the intention of this study to authentically convey the participants' experiences and perceptions.

### 4.3. Future directions

Based on the results and limitations of the study, several directions for future research present themselves. Firstly, future researchers should validate these results and the model of the Liberatory process engendered by engagement with Africentric knowledge. Given today's increasing emphasis on racial identity, finding educational institutions with curricula that engage with that ever-growing knowledge base should become easier with the passage of time. Secondly, this model of the Liberatory process might be explored through research in other educational contexts. Future researchers might involve themselves in research that enables their own students to engage with Africentric intellectual knowledge and record the results. This would indicate if the same Liberatory process applies at a lower level of education, or if the more reflective nature of graduate education was an outsized factor in this study. Finally, conducting a truly comprehensive review of the literature regarding racial identity in adult education might prove valuable to understand where the field currently stands and what is required to advance this intellectually and practically significant line of research.

## Data availability statement

The original contributions presented in the study are included in the article/supplementary material, further inquiries can be directed to the corresponding author.

## Ethics statement

Ethical review and approval was not required for the study on human participants in accordance with the local legislation and institutional requirements. The patients/participants provided their written informed consent to participate in this study.

## Author contributions

The author confirms being the sole contributor of this work and has approved it for publication.
